# Musical Collaboration in Rhythmic Improvisation

**DOI:** 10.3390/e22020233

**Published:** 2020-02-19

**Authors:** Shinnosuke Nakayama, Vrishin R. Soman, Maurizio Porfiri

**Affiliations:** 1 Department of Mechanical and Aerospace Engineering, New York University Tandon School of Engineering, 6 MetroTech Center, Brooklyn, NY 11201, USA; sn2286@nyu.edu (S.N.); vrs283@nyu.edu (V.R.S.); 2 Department of Biomedical Engineering, New York University Tandon School of Engineering, 6 MetroTech Center, Brooklyn, NY 11201, USA

**Keywords:** collaboration, information theory, music, recurrence, symbolic dynamics

## Abstract

Despite our intimate relationship with music in every-day life, we know little about how people create music. A particularly elusive area of study entails the spontaneous collaborative musical creation in the absence of rehearsals or scripts. Toward this aim, we designed an experiment in which pairs of players collaboratively created music in rhythmic improvisation. Rhythmic patterns and collaborative processes were investigated through symbolic-recurrence quantification and information theory, applied to the time series of the sound created by the players. Working with real data on collaborative rhythmic improvisation, we identified features of improvised music and elucidated underlying processes of collaboration. Players preferred certain patterns over others, and their musical experience drove musical collaboration when rhythmic improvisation started. These results unfold prevailing rhythmic features in collaborative music creation while informing the complex dynamics of the underlying processes.

## 1. Introduction

Across cultures in history, music has always been a universal part of human life [1]. Whether pursued as a form of art to express ourselves or as a therapeutic tool to address emotional, cognitive, physical, and social needs, we are all familiar with the nature and value of music [2]. However, little is known about the process of creating music, even in simple rhythmic improvisation comprising a few notes.

Igor Stravinsky stated that a musical form is “far closer to mathematics than to literature—not perhaps to mathematics itself, but certainly to something like mathematical thinking and mathematical relationships” [3]. For example, Ernîo Lendvai identified the presence of Fibonacci numbers and golden ratios in many of Béla Bartók’s pieces [4]. Musical structures can be visualized and quantified by studying self-similarity over time from recurrent patterns [5,6,7] or constructing networks on the basis of the pitch and duration of notes [8]. Interestingly, recurrence-quantification analysis revealed that compositions of Bach’s inventions and sinfonias are more complex than a mere Markov process [9]. Predictably, the mathematical elements of music can be uncovered through machine learning, which could be used to detect the temporal structure of music [10], and even to compose music [11,12].

From mathematically principled analysis of musical structures within a single piece, one may attempt to compare pieces by different musicians. For example, a popular approach to the comparison of musical structures is to measure the distance between recurrence plots constructed on musical features [13]. Although the approach could, in principle, be extended to the study of musical collaboration, the literature in this field is scarce. To the best of our knowledge, the application of recurrence-quantification methods to musical collaboration is limited to Walton et al. [14], who evaluated the dependency of two acoustic signals in collaborative music creation through cross-recurrence quantification. The area of spontaneous synchronization of beats shares some similarities with musical creation [15,16], but music is generally more complex than synchronization on an emerging pattern.

A unique setting to experimentally study musical collaboration is improvisation, where music spontaneously emerges from unstructured dynamical interactions between players who embrace a sequence of decisions toward their sense of music [17]. Without rehearsals or scripts, music can be created through cognitive efforts that involve short- and long-term memory [18], and communication based on calls and responses [19]. In this context, understanding the processes and outcomes of musical improvisation may offer a deeper insight into human nature of musical cognition. However, little effort has been directed toward the application of mathematically principled approaches to elucidate how people interact during improvisation and what kind of music they create.

In this study, we investigated processes and outcomes of collaborative musical improvisation. We focused on situations where people without professional training create music together through rhythm, which constitutes a fundamental element of music that humans are wired to appreciate [20,21]. In the experiment, participants with various musical expertise were randomly paired and asked to freely create music without rehearsals or scripts. Facing against each other in a room, each participant was provided with a velocity-sensitive drum pad with only two marimba notes. In two improvisation sessions, participants were allowed to interact only through the music they heard and created, thereby eliminating visual cues that may otherwise contribute to musical collaboration [22,23]. In this sense, the outcomes of the collaboration were also the means that supported the processes of collaboration through the sharing and transfer of information.

By examining the sound data collected in the experiment, we studied the rhythmic patterns of improvised music through recurrence quantification, which offers a mathematically principled approach for studying musical structures [7,13]. From the percussive sound produced by the two players, we formed a symbolic time series where each symbol identified a specific ordinal pattern in the amplitude of consecutive sound samples. Each time series was examined through the lens of recurrence-quantification analysis to create symbolic-recurrence plots that captured the recurrences of different ordinal patterns in a multidimensional recurrence plot [24,25]. The more the points that populated the recurrence plot, the more repetitive the rhythm was and the higher the symbolic-recurrence rate was. Entropy on symbolic recurrences was used to quantify preference for specific musical patterns that emerged during collaboration. Hence, low entropy values indicated a preference of the players for specific rhythmic patterns, while larger entropy values pertained to a less marked preference for patterns over others. We hypothesize the emergence of recurring patterns with a potential preference for specific musical patterns, as found in human solo drumming [26].

To elucidate the interaction between the players, we performed multivariate recurrence analysis on the two time series of the sound amplitudes produced by the players within each pair. From these time series, we measured the amount of information that was shared and transferred between the two players through salient information-theoretic metrics on joint symbolic-recurrence plots [25,27]. Mutual information was used to quantify the association between the rhythmic patterns of the two players, and transfer entropy was employed to measure the responsiveness of the players to their partners. We hypothesize that the process of collaboratively and spontaneously creating music is supported by strong information sharing and transfer between the players.

To explain the variation in the degree of interaction between the players, we inspected the expertise of the participants in playing music, acquired through independent surveys. Following the mental model on teamwork that emphasizes the importance of individual experience and skills on the outcome of collaboration [28], we hypothesize that the extent of information sharing and transfer within a pair is explained by the musical expertise of the pair.

## 2. Materials and Methods

### 2.1. Experiment Setup

The instruments provided to the participants were MIDI controllers with pads (nanoPAD2, KORG, Melville, NY, USA), digitally programmed with samples of a marimba sourced from the public-domain library of the University of Iowa Electronic Music Studios. The MIDI controllers fed velocity-sensitive information directly to the recording interface via two sets of adjacent rubber pads. The recording interface was a standard digital-audio-production application (REAPER, Cockos Incorporated, New York, NY, USA). This platform was chosen because of the customizable nature of the interface, audio-routing capabilities, and compatibility with audio drivers and the MIDI instruments. The recordings were taken at 44,100 Hz on a Windows laptop augmented with an external USB sound card in addition to inbuilt audio capabilities.

Within the REAPER interface, incoming MIDI signals were rendered as the sampled marimba audio signal and sent to both participants’ sets of headphones (ATH-AVC200, Audio-Technica, Tokyo, Japan). The MIDI controller was configured to play a set of two notes. MIDI recordings were performed in REAPER and saved as REAPER project files. Data were exported as WAV audio files for listening and further analysis.

Two players controlled four notes in an F-major seventh chord (F, A, C, and E), one of the traditional chords in Western music that is frequently utilized within an improvisational context [29]. To promote collaboration, each player could only make a partial chord on their own, which would then be extended with the addition of their partners’ complementary notes. Specifically, one player was assigned the fifth and seventh (C and E) of the chord, while the other player was assigned the first and third (F and A). This selection was also helpful for players to discriminate their sound from that of their partners. Although F and E are dissonant, our participants showed preference for these keys over the middle ones (A and C; see Appendix A).

### 2.2. Data Collection

**Figure 1:**
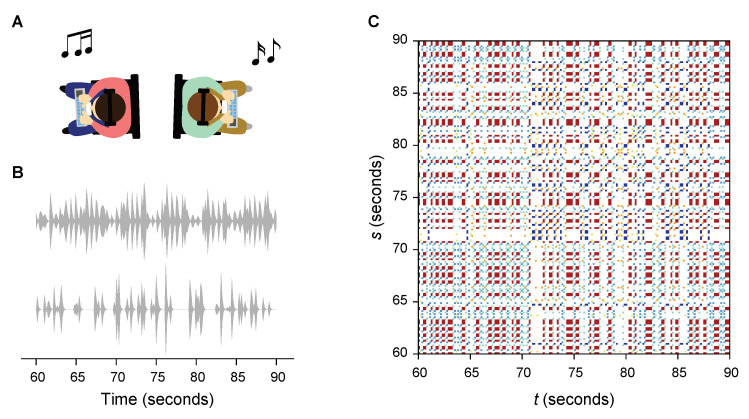
Study flow. (**A**) Two participants sit facing against each other in a same room to create music together by improvising using drum pads while acoustically communicating with each other through headphones. (**B**) Sound amplitudes extracted (2 min × 2 sessions, excluding first 15 s with a backing track from each session). (**C**) Music recurrence plots created from sound amplitudes by symbolizing following ordinal patterns. Colored areas of a recurrent plot indicate recurrence of a symbol at time *t* and *s*, with colors representing which symbol recurred. Portion of the recurrence plot shown for clarity.

Participants were recruited from the New York University community in the Brooklyn campus, NY, USA. Each trial consisted of a tutorial followed by two experiment sessions. The tutorial was based off a classic one-note call-and-response exercise, toward introducing a standardized basis of collaboration and improvisation. Two participants sat in the same room facing away from each other (Figure 1). The headphones of the participants were connected to two distinct audio outputs, and audio information was isolated between participants during the tutorial. Through the headphone, the participants heard a short series of measure-long rhythms, each followed by two measures of rest, progressively increasing in complexity. The experimenter instructed them to mirror what they heard exactly by using their respective base notes. Then, the participants were exposed to the same series of rhythms through their headphones and instructed to improvise a response instead of merely repeating. The tutorial ended with a 30 s practice session where they could use both the notes while listening to a prerecorded drum backing track.

During a short intermission after the tutorial, participants were asked to complete a survey regarding their musical expertise. Specifically, they filled out their experience in playing music with others on the Likert scale (0: never, 1: rarely, 2: sometimes, 3: frequently, and 4: always) and the duration of practicing music in years.

Upon completing the surveys, the participants began the experiment that consisted of two improvisation sessions, each of 2 min in duration. Before commencing the session, the experimenter instructed the participants as follows: “Now you will be playing together and collaborating in your improvisation. Feel free to experiment, but remember to collaborate”. Different from the tutorial, the participants’ headphones were connected to a single audio outport on the USB sound card by using an audio-splitter device so that they would hear the music they collaboratively created. The same drum backing track used in the tutorial was played for the first 15 s of each session, providing a starting tempo for the participants. After these initial 15 s, there was no accompaniment, and the participants improvised for the duration of each 2 min session. Between sessions, the notes controlled by each participant were swapped to randomize the key assignment. In total, we collected data from 30 pairs. The experiment was approved by the Institutional Review Board of the University (IRB-FY2017-898).

### 2.3. Symbolic-Recurrence Quantification

Given scalar time series ${\left\{x_{t}\right\}}_{t=1}^{T}$ of *T* samples, we constructed the symbolic time series of m! symbols on the basis of ordinal patterns of length *m*, ${\left\{S^{x}\left({\bar{x}}_{t}\right)\right\}}_{t=1}^{\bar{T}}$, where $\bar{T}=T-m+1$, ${\bar{x}}_{t}=\left(x_{t},x_{t+1},\ldots ,x_{\bar{T}}\right)$ is the phase-space vector at time *t*, and $S^{x}\left(\cdot \right)$ is symbolization mapping. For example, if m=3, we had an alphabet $\Gamma^{x}$ of six symbols, each identifying a specific pattern for three consecutive readings in the time series from a sequence of three numbers that continuously decreased to three that instead steadily increased. From the symbolic time series, we assembled a symbolic-recurrence plot [24] to encode the recurrence of each symbol of the alphabet in time (Figure 1), that is,

(1) $$SR_{ts}^{x}\left(\pi^{x}\right)=\left\{\begin{array}{cc} 1 & \mathrm{if}\;S^{x}\left({\bar{x}}_{t}\right)=S^{x}\left({\bar{x}}_{s}\right)=\pi^{x},\\ 0 & \mathrm{otherwise}. \end{array}\right.$$

The symbolic-recurrence rate of generic symbol $\pi^{x}$ was computed by counting the total fraction of recurring symbol, that is,

(2) $$SRR\left(\pi^{x}\right)=\frac{1}{\bar{T}\left(\bar{T}-1\right)}\sum_{\begin{array}{c} t,s=1\\ t\neq s \end{array}}^{\bar{T}}SR_{ts}^{x}\left(\pi^{x}\right).$$

This quantity estimated the probability of recurrence of $\pi^{x}$. By summing these partial rates, we calculated symbolic-recurrence rate SRR that measured the overall extent of recurrence without discriminating whether it pertained to few or many symbols that were repeating in time. For reference, an independent identically distributed time series would have a symbolic-recurrence rate of 1/m!.

To afford further recurrence quantification in the phase space, we examined the entropy of the symbolic-recurrence plot [27]. By exclusively focusing on the portion of the recurrence plot that encoded recurrence, we estimated the probability of recurrence of generic symbol $\pi^{x}$ as the fraction of its recurrences over the total number of recurrences, that is,

(3) $$P^{x}\left(\pi^{x}\right)=\frac{SRR(\pi^{x})}{SRR}.$$

Hence, the entropy of time series ${\left\{x_{t}\right\}}_{t=1}^{T}$ upon symbolization is

(4) $$H\left(x_{t}\right)=-\sum_{\pi^{x}\in \Gamma^{x}}P^{x}\left(\pi^{x}\right)\log P^{x}\left(\pi^{x}\right),$$

where we used the logarithm to base 2 so that we measured entropy in “bits.” Again, for an independent identically distributed time series, entropy should be log(m!).

When looking at two or more time series, we can study a multivariate form of the symbolic-recurrence plot in which we examine a phase-space vector in the higher dimensional space given by the Cartesian product of the original phase spaces. In this vein, the symbolic-recurrence plot of two time series ${\left\{\left(x_{t},y_{t}\right)\right\}}_{t=1}^{T}$ with ordinal patterns of length *m* tracks ${\left(m!\right)}^{2}$ symbol pairs. From this symbolic-recurrence plot, we computed the mutual information between time series ${\left\{x_{t}\right\}}_{t=1}^{T}$ and ${\left\{y_{t}\right\}}_{t=1}^{T}$ upon symbolization as

(5) $$I^{xy}=H\left(x_{t}\right)+H\left(y_{t}\right)-H\left(x_{t},y_{t}\right),$$

where $H(x_{t},y_{t})$ is the joint entropy of ${\left\{x_{t}\right\}}_{t=1}^{T}$ and ${\left\{y_{t}\right\}}_{t=1}^{T}$ upon symbolization. Similarly, we could examine the symbolic-recurrence plot of multivariate time series ${\left\{\left(x_{t+1},x_{t},y_{t}\right)\right\}}_{t=1}^{T-1}$ to compute transfer entropy on symbolic recurrences as

(6) $$TE^{y\to x}=H\left(x_{t+1}|x_{t}\right)-H\left(x_{t+1}|x_{t},y_{t}\right),$$

where $H(x_{t+1}|x_{t})$ is the entropy of ${\left\{x_{t+1}\right\}}_{t=1}^{T-1}$ conditional to ${\left\{x_{t}\right\}}_{t=1}^{T-1}$, and $H(x_{t+1}|x_{t},y_{t})$ is the entropy of ${\left\{x_{t+1}\right\}}_{t=1}^{T-1}$ conditional to ${\left\{\left(x_{t},y_{t}\right)\right\}}_{t=1}^{T-1}$, upon symbolization. Analogous to transfer entropy on two univariate time series [30], transfer entropy on symbolic recurrences measures the reduction of uncertainty in predicting the future state of one time series given the current state of the other time series within a probability space constructed over symbolic recurrences [27]. With respect to focal time series ${\left\{x_{t}\right\}}_{t=1}^{T}$, this value quantified the directional influence of other time series ${\left\{y_{t}\right\}}_{t=1}^{T}$.

Throughout the study, we downsampled the time series at a rate of 150 ms, mirroring typical auditory reaction time [31]. This yielded a total of T=800 samples for each trial. To capture the complexity of the time series while balancing the limited length of the time series, we used m=3 for symbolization. In the appendix, we illustrate the robustness of these choices by examining the cases of downsampling at 100 ms with m=3 and downsampling at 150 ms with m=2.

### 2.4. Analysis

To test whether musical improvisation brings about an emergence of recurring patterns with marked preference for certain patterns, we compared the SRR and entropy of the music created by pairs against random values. To that end, we created a new dataset of 30 pairs by randomly pairing individual sound data of the 60 participants for each session. Hence, the new dataset represented music created by pairs where players within a pair could not acoustically communicate with each other. The observed mean of each variable was compared against the corresponding null distribution of the mean, which was generated by repeating the shuffling process 20,000 times. When the observed mean fell outside of a 95 percentile of null distribution (two-sided), we deemed that the variable was significantly different from a random one.

Similarly, we tested whether players exhibited a greater extent of information sharing and transfer within pairs by comparing the mean mutual information of 30 pairs and the transfer entropy of 60 players against random values. To that end, we generated the null distribution of the mean of each variable for each session by randomly pairing individual sound data and repeating the process 20,000 times in the same way as described above. When the observed mean fell outside of a 95 percentile of null distribution (one-sided), we deemed that the variable was significantly different from a random one.

Further, we investigated the difference between sessions and consistency within pairs in the musical characteristics and extents of information sharing and transfer. Specifically, SRR, entropy, mutual information, and transfer entropy were compared between sessions by using a paired *t*-test. Similarly, within-pair consistency in these values were investigated using Pearson’s correlation.

Next, we investigated the musical expertise of each player as a possible factor for variation in the extent of information sharing and transfer among pairs. For information sharing, we characterized pair traits with the sum of experience in playing music with others (score 0–8) and the difference (score 0–4), as well as the sum and difference of music-practicing duration (in years). Mutual information was fitted into a generalized linear model with gamma error distribution and a log link. The interaction terms of the sum and difference were also included in the model. For information transfer, transfer entropy that focal players received from their partners was fitted into a generalized linear model, with the musical expertise of a focal player and their partner as explanatory variables. The model was specified with gamma error distribution and a log link. The interaction terms of focal players and their partners’ musical expertise were also included in the model.

All data analyses were performed using base R ver. 3.6.0 [32], R package ‘seewave’ ver. 2.1.4 [33], ‘car’ ver. 3.0-3 [34], and Python package ‘NumPy’ ver. 1.17.2 [35].

## 3. Results and Discussion

### 3.1. Symbolic-Recurrence Quantification of Music

**Figure 2:**
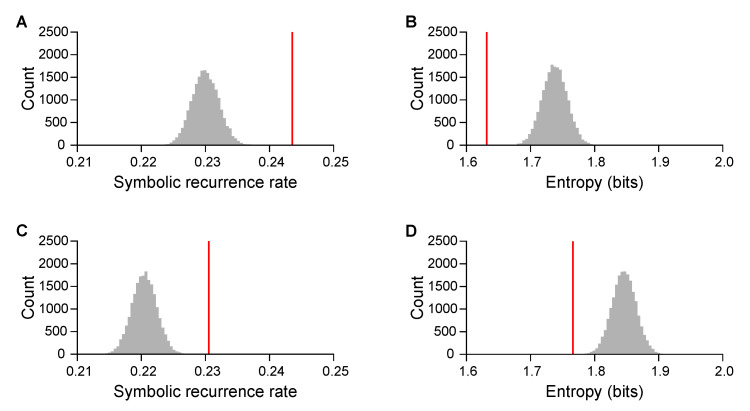
Mean observed recurrence metrics of the music created by a pair against null distributions: music was characterized by rhythmic patterns, and players preferred some patterns over others. First session: (**A**) symbolic-recurrence rate and (**B**) entropy. Second session: (**C**) symbolic-recurrence rate and (**D**) entropy. Vertical red lines, observed means; grey areas, null distributions of means.

For each of the two improvisation sessions, we characterized the music created by each pair in terms of symbolic-recurrence rate (SRR) and entropy. We observed an SRR of 0.244±0.034 (mean ± standard deviation, N=30 pairs) in the first session, and of 0.231±0.033 in the second one. The mean of SRR was significantly greater than chance (two-sided permutation test, p<0.001 for both sessions; Figure 2). Overall, pairs showed consistent values of SRR between the sessions (Pearson’s correlation, r=0.659,t=4.638,df=28,p<0.001), but values were smaller in the second session (paired *t*-test, t=2.544,df=29,p=0.017). The recordings of the experiments with the lowest and the highest SRR are available at https://github.com/shinn1/music.

The entropy of the music was 1.632±0.324 bits in the first session (N=30 pairs), and 1.766±0.309 bits in the second one. The mean of the entropy was significantly smaller than chance (p<0.001 for both sessions; Figure 2). Entropy was correlated between sessions (r=0.693,t=5.085,df=28,p<0.001), although pairs showed greater values in the second session (t=2.913,df=29,p=0.007).

An empirical study demonstrated common structural regularities in rhythm when humans solo-play a drum [26]; our results revealed the emergence of such regularities in collaborative music creation. Improvised music collaboratively created by our participants was characterized by repetitive rhythmic patterns with marked preference for specific patterns over others, indicated by higher symbolic-recurrence rates and lower entropy. Considering that the origin of music is rooted in social activities [36,37], humans may have an innate inclination to rhythmic patterns that are easy to learn and memorize [26]. Indeed, people are more likely to perceive rhythmic patterns as a division of sound duration by small integers [38]. Cross-cultural similarities in rhythmic patterns [39,40] further support the possibility. Unlike solo music, however, musical collaboration through improvisation requires the social exchanges of musical motifs with dynamic responses and adjustments [17,19,41]. The need for such complex interaction might be the reason why children are incapable of performing collaborative improvisation in music until later in life [42]. Our results confirmed that adults are able to exchange musical motifs through acoustic cues toward collaboratively creating music.

### 3.2. Information Sharing and Transfer on Symbolic Recurrence

**Figure 3:**
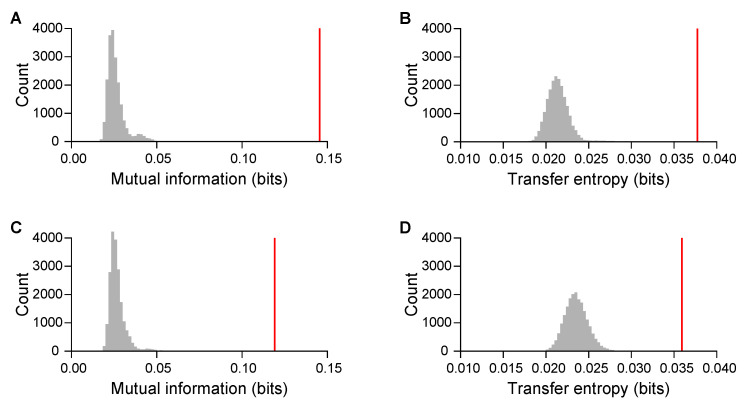
Mean observed recurrence metrics of interaction within a pair against null distributions: process of musical collaboration is underpinned by information sharing and transfer between players. First session: (**A**) mutual information and (**B**) transfer entropy received from partners. Second session: (**C**) mutual information and (**D**) transfer entropy received from partners. Vertical red lines, observed means; grey areas, null distributions of means.

How players shared information with each other and how they responded to their partners were measured through mutual information and transfer entropy on symbolic recurrences, respectively. For each trial, we computed one value of mutual information and two values of transfer entropy (from partner to focal player, corresponding to the responsiveness of the focal player). We observed mutual information of 0.145±0.160 bits in the first session (N=30 pairs), and of 0.119±0.129 bits in the second. The mean of mutual information was significantly greater than chance (permutation test, p<0.001 for both sessions; Figure 3). Mutual information was similar between sessions (t=1.005,df=29,p=0.323), and pairs showed strong consistency across sessions (r=0.539,t=3.389,df=28,p=0.002). The recordings of the experiments with the lowest and highest mutual information are available at https://github.com/shinn1/music.

Transfer entropy was 0.038±0.036 bits in the first session (N=60 players), and 0.036±0.024 bits in the second. Again, the mean was significantly greater than chance (p<0.001 for both sessions; Figure 3), and values were correlated between sessions (r=0.278,t=2.201,df=58,p=0.032). There was no change between sessions (t=0.373,df=59,p=0.710).

### 3.3. Effects of Pair and Individual Traits on Information Sharing and Transfer

The survey revealed a wide range of musical expertise between participants, measured through two independent variables. With respect to experience in playing music with others, 12 participants answered “never” (score 0), 11 “rarely” (1), 17 “sometimes” (2), 13 “frequently” (3), and 7 “always” (4). The duration of practicing music ranged from 0 to 15 years (first quartile: 0, second: 3, and third: 7 years).

**Figure 4:**
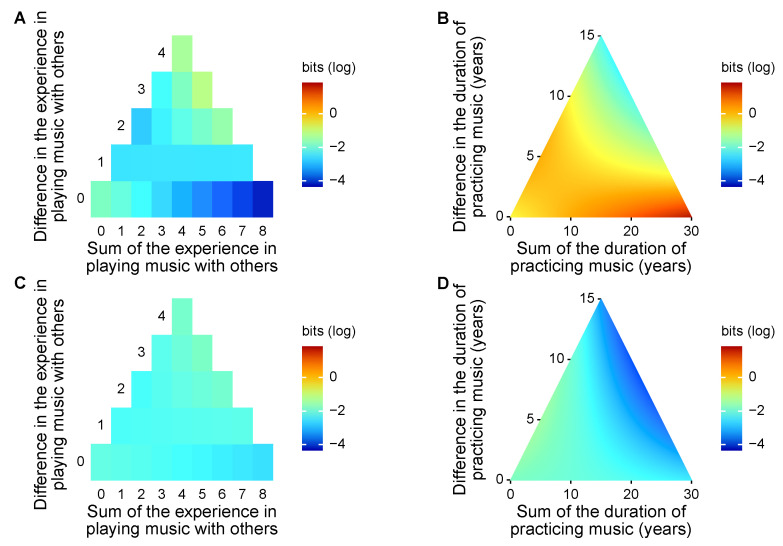
Effects of pairwise traits in musical expertise on mutual information: in the first session, information sharing was favored by differences in experience in playing with others and similarities in duration of practicing music. First session: (**A**) experience in playing music with others and (**B**) duration of practicing music. Second session: (**C**) experience in playing music with others and (**D**) duration of practicing music.

Delving into variations in information sharing and transfer across trials, we confirmed our hypothesis that players’ expertise in playing music is responsible for the processes of musical collaboration. Musical expertise explained variation in mutual information in pairs in the initial phase of the improvised musical collaboration (Figure 4). In the first experiment session, participants were found to share more information when playing music with partners that had a different level of experience in musical collaboration.

Specifically, in the first session, mutual information was associated with the interaction between the within-pair sum of experience in musical collaboration and within-pair difference ($\chi_{1}^{2}=6.664,p=0.010$). It was also marginally explained by the interaction between the within-pair difference in duration of practicing music and within-pair difference ($\chi_{1}^{2}=3.507,p=0.061$) and by the within-pair difference ($\chi_{1}^{2}=3.030,p=0.082$), but not by the within-pair sum ($\chi_{1}^{2}=0.643,p=0.423$). These results indicated that pairing experts with novices in musical collaboration favored information sharing compared to pairing players with moderate experience in musical collaboration. By contrast, similarities within the pair in the duration of practicing music were conducive to information sharing, although pairing experts in musical instruments led to stronger information sharing than pairing novices.

In the second session, however, mutual information was not explained by experience in musical collaboration ($\chi_{1}^{2}=0.054,p=0.817$ for the sum; $\chi_{1}^{2}=0.256,p=0.613$ for the difference; $\chi_{1}^{2}=0.497,p=0.481$ for the interaction). The duration of practicing music did not explain the variation in mutual information, either ($\chi_{1}^{2}=2.199,p=0.138$ for the sum; $\chi_{1}^{2}=0.042,p=0.837$ for the difference; $\chi_{1}^{2}=0.975,p=0.323$ for the interaction).

**Figure 5:**
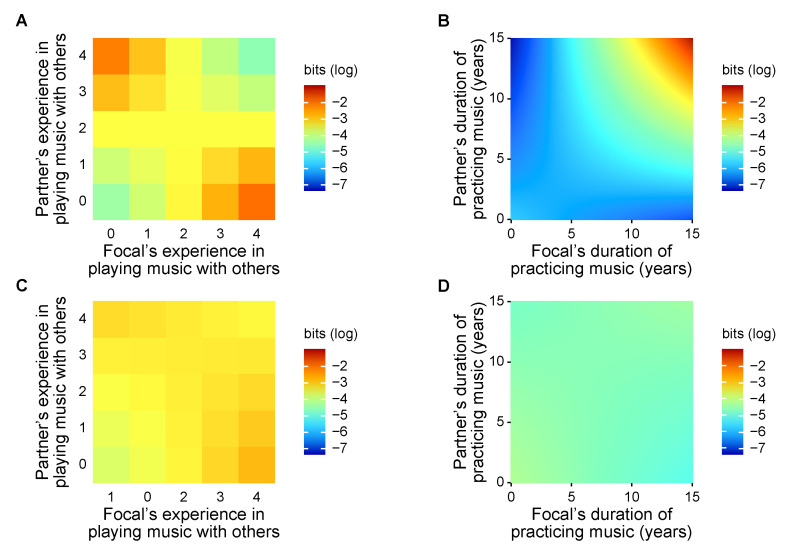
Effects of individual traits in musical expertise on transfer entropy (from partner to focal player): in the first session, information transfer was favored by differences in experience in playing with others and similarities in duration of practicing music. First session: (**A**) experience in playing music with others and (**B**) duration of practicing music. Second session: (**C**) experience in playing music with others and (**D**) duration of practicing music.

Variation in information sharing was partly associated with how individuals musically responded to their partners, quantified through transfer entropy on symbolic recurrence. In the first session, transfer entropy was associated with the interaction between focal player and partner in their experience in musical collaboration ($\chi_{1}^{2}=13.465,p<0.001$), and in the duration of practicing music ($\chi_{1}^{2}=21.467,p<0.001$; Figure 5). These results indicated that, in the initial phase of the musical collaboration, players’ responses to their partners were explained by the musical expertise of both players. Predictably, novices to musical collaboration are influenced by partners who have experience in playing with others; these experienced partners can, in turn, adjust their rhythm more when playing with novices. The extent of this feedback depends on their relative training in music, whereby participants responded more strongly when partnered with others who practiced music for a similar duration. In this way, participants adjusted acoustic responses to their partner without knowing their musical expertise.

By contrast, in the second session, transfer entropy was not explained by experience in musical collaboration ($\chi_{1}^{2}=1.906,p=0.167$ for focal players; $\chi_{1}^{2}=0.498,p=0.480$ for partners; $\chi_{1}^{2}=0937,p=0.333$ for the interaction). The duration of practicing music did not contribute to the model fit ($\chi_{1}^{2}=0.620,p=0.431$ for partners; $\chi_{1}^{2}=0.877,p=0.349$ for the interaction), with marginal significance for the focal players’ duration of practicing music ($\chi_{1}^{2}=3.592,p=0.058$).

## 4. Conclusions

This is the first study that elucidated the processes and outcomes of collaborative musical improvisation through a mathematically principled approach. Pairs created music characterized by repetitive rhythmic patterns with marked preference for specific patterns over others, and the formation of such musical characteristics was underpinned by information sharing and transfer between players. Musical collaboration was established in the initial phase through players’ musical expertise, but the influence of musical expertise disappeared over time. These results unfolded prevailing rhythmic features in collaborative music creation while informing the complex dynamics of the underlying processes.

Music created by the pairs evolved over time, where rhythms became less repetitive, with more diverse patterns. These musical traits suggest that participants attempted to invent new rhythmic patterns once they established communication, resulting in the creation of music that was more unpredictable. Although the extent of information sharing and transfer in the second session was correlated with those in the first session, the musical expertise of the players no longer explained the variations. One possibility is that musical expertise played a role only until participants understood their partner’s rhythmic inclinations and responses through learning [19]. We also propose that, as time progressed, players gained confidence in their own musical expression, living a unique moment of inspiration independent of their musical expertise or that of their partner. Further study is needed to fully understand the dynamics of improvised music over time and the underlying factors that contribute to the dynamics.

In this study, we did not appraise the quality of the improvised music, as the notion of music is elusive [36]. Although most music entails common traits in rhythms, such as a use of isochronous beats and a metrical hierarchy in meters [39], music perception is largely shaped by enculturation [43,44,45,46]. Hence, people from different cultural backgrounds may exhibit disparate preferences [47,48,49,50]. For example, American infants prefer drum patterns with familiar Western meters (pulse duration ratio of 2:1:1) over unfamiliar Balkan meters (pulse duration ratio of 3:2:2), whereas Turkish infants who are familiar with both meters do not express preference [51]. Considering that participants in our study were from a student pool of a university that is home to students from diverse cultures, similarity in cultural backgrounds could also explain the extent of information sharing and transfer, in addition to their musical expertise.

In conclusion, we studied the processes and outcomes of musical collaboration in rhythmic improvisation through symbolic-recurrence quantification and information theory. In reality, musical collaboration could be achieved through other elements of music, such as melody, harmony, timber, and texture [52]. Further, there exist implicit rules that facilitate musical collaboration in jam sessions [53,54,55], including body gestures [23]. Nevertheless, our results shed light on the human ability of musical collaboration through rhythm, which constitutes a fundamental element of music from evolutionary and ethnomusicological perspectives [56,57].

## Figures and Tables

**Figure A1:**
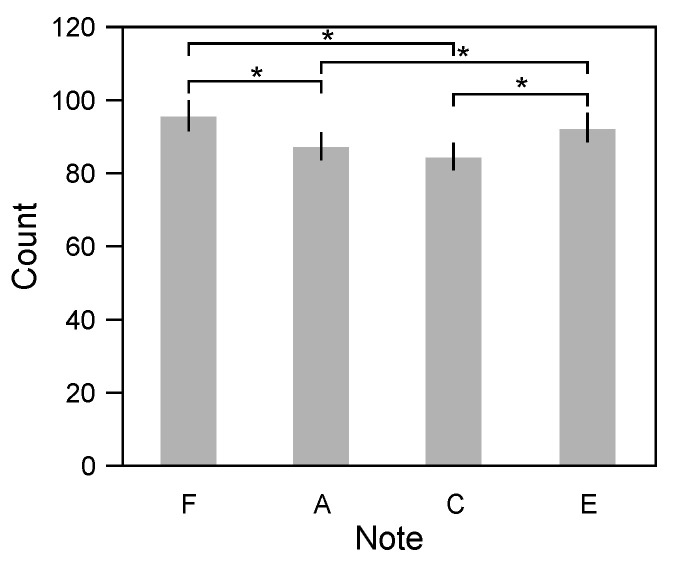
Frequency of notes played by participants. Asterisks indicate significant difference at a 0.05 level.

**Figure A2:**
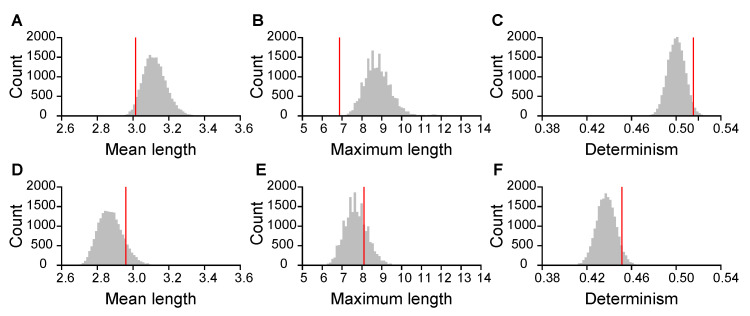
Diagonal lines of symbolic-recurrence plots. Mean length, maximal length, and determinism in (**A**–**C**) first and (**D**–**F**) second session. Vertical red lines, observed means; grey areas, null distributions of means.

**Figure A3:**
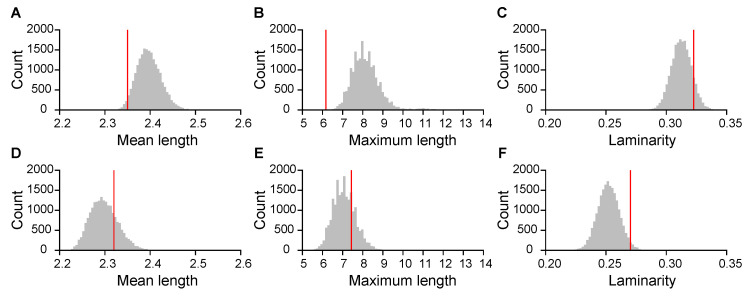
Vertical lines of symbolic-recurrence plots. Mean length, maximal length, and laminarity in (**A**–**C**) first and (**D**–**F**) second session. Vertical red lines, observed means; grey areas, null distributions of means.

**Figure A4:**
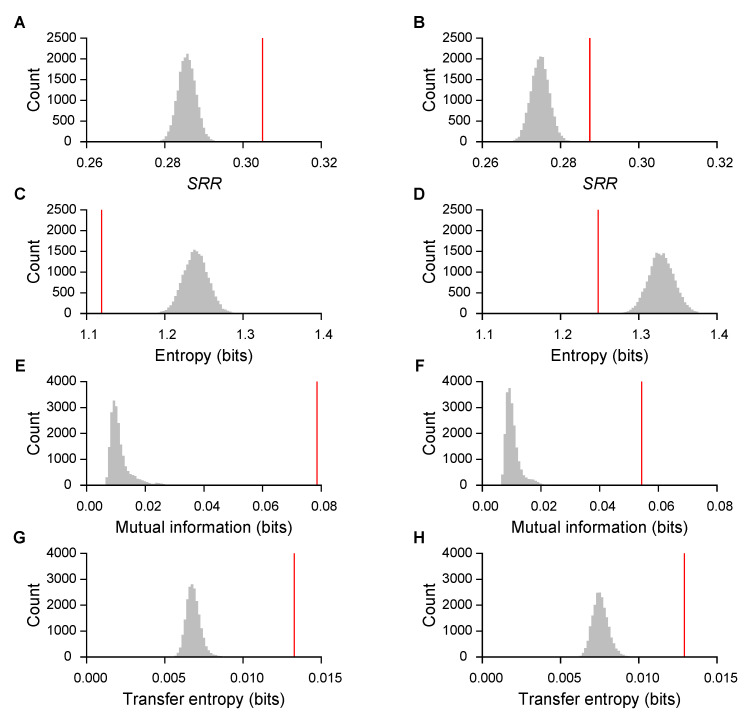
Mean observed recurrence metrics of music created by a pair and the interaction within the pair for a downsampling rate of 100 ms against null distributions. Symbolic-recurrence rate in (**A**) first and (**B**) second session, entropy in (**C**) first and (**D**) second session, mutual information in (**E**) first and (**F**) second session, and transfer entropy in (**G**) first and (**H**) second session. Vertical red lines, observed means; grey areas, null distributions of means.

**Figure A5:**
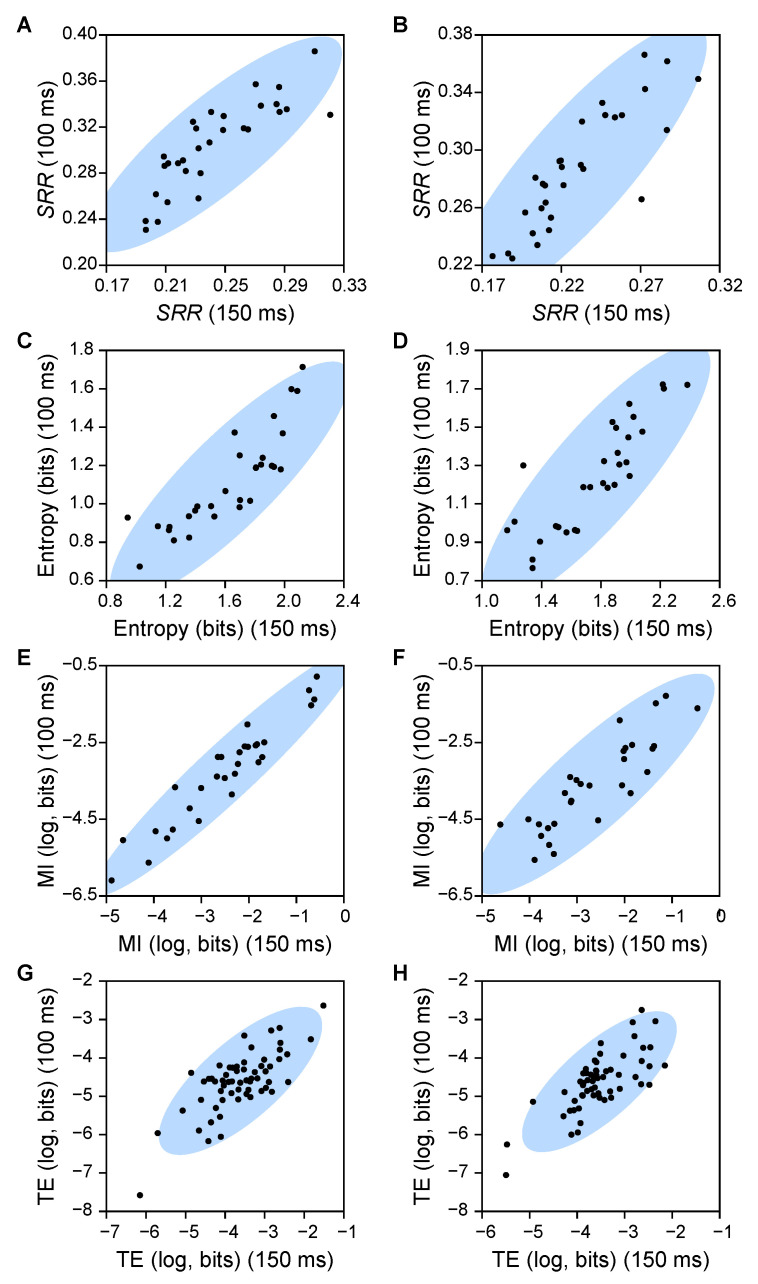
Correlations between the original recurrence metrics down-sampled at a 150-ms interval and those down-sampled at a 100-ms interval. (**A**) Symbolic-recurrence rate in the first session and (**B**) in the second session, (**C**) entropy in the first session and (**D**) in the second session, (**E**) mutual information in the first session and (**F**) in the second session, and (**G**) transfer entropy in the first session and (**H**) in the second session. Ellipses represent 95% confidence areas.

**Figure A6:**
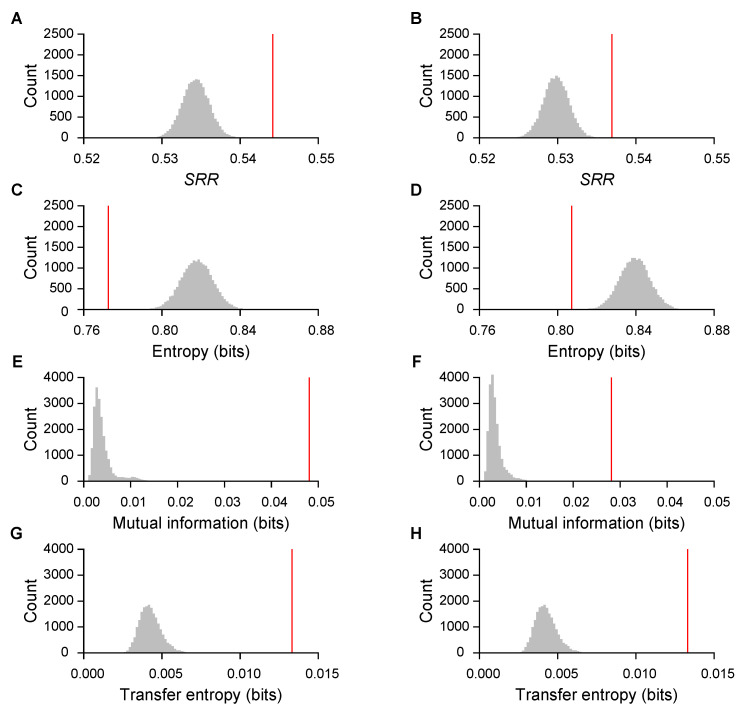
Mean observed recurrence metrics of music created by a pair and the interaction within the pair symbolized with m=2, against null distributions. Symbolic-recurrence rate in (**A**) first and (**B**) second session, entropy in (**C**) first and (**D**) second session, mutual information in (**E**) first and (**F**) second session, and transfer entropy in (**G**) first and (**H**) second session. Vertical red lines, observed means; grey areas, null distributions of means.

**Figure A7:**
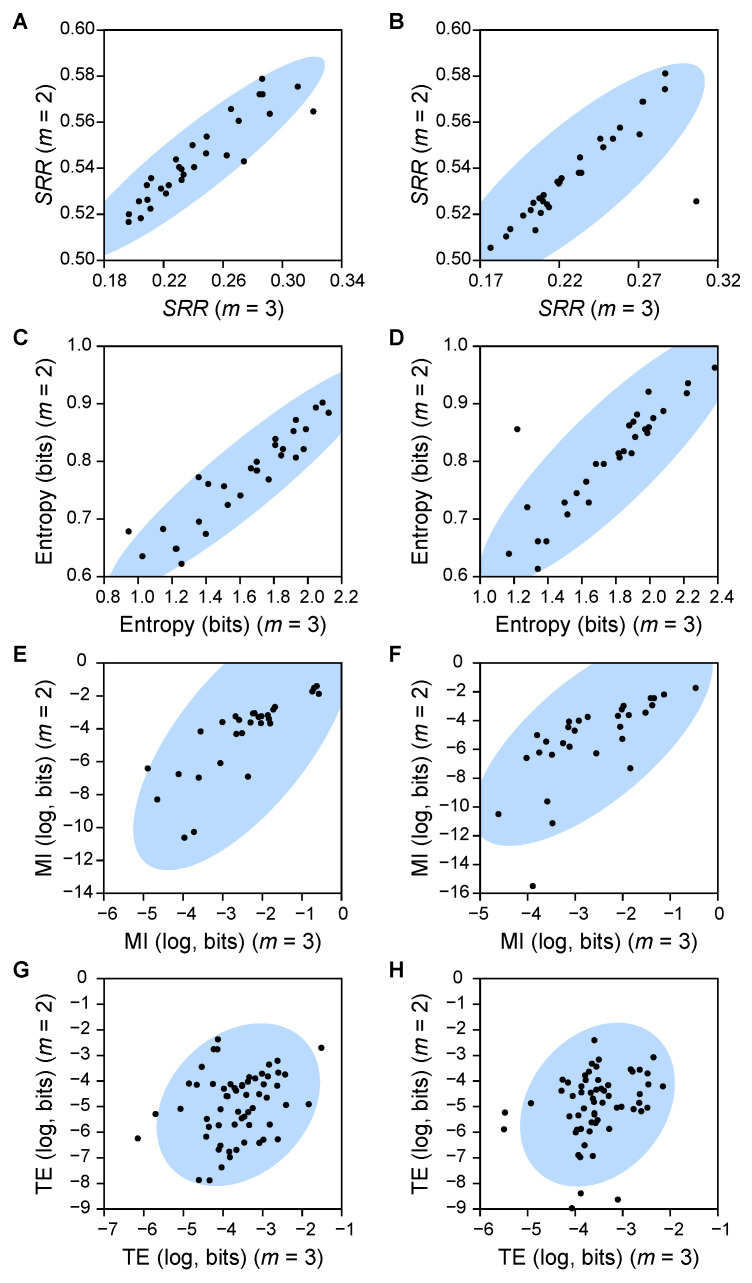
Correlations between original recurrence metrics symbolized with m=3 and those with m=2. Symbolic-recurrence rate in (**A**) first and (**B**) second session, entropy in (**C**) first and (**D**) second session, mutual information in (**E**) first and (**F**) second session, and transfer entropy in (**G**) first and (**H**) second session. Ellipses represent 95% confidence areas.

## Appendix A

### Appendix A.1. Preference of Notes Played by Participants

To investigate whether participants preferred one of the notes over the other during the collaborative musical improvisation, we investigated the frequency of the notes. From the MIDI data, we counted the occurrence of each key press event (F, A, C, and E) for each participant. Difference in note frequency was tested by fitting into a generalized linear mixed-effect model, specifying Poisson errors with a log link and participant identity as a random effect, followed by a likelihood-ratio test and Tukey’s post hoc test. Statistical analyses were performed using R packages ‘lme4’ ver. 1.1-21 [58], ‘car’ ver. 3.0-3 [34], and ‘multcomp’ ver. 1.4-10 [59].

There was an overall difference in note frequency ($\chi_{3}^{2}=52.461,p<0.001$; Figure A1). During the two sessions, the participants pressed F 100.5±53.1 times (mean ± standard deviation), A 91.7±50.8 times, C 88.8±44.1 times, and E 97.1±51.5 times. The occurrence of E was more frequent than that of A (z=3.036,p=0.013) and C (z=4.708,p<0.001). Similarly, F was observed more frequently than A (z=4.900,p<0.001) and C (z=6.569,p<0.001). There was no difference in the frequencies of C and A (z=1.673,p=0.338), and of F and E (z=1.866,p=0.243). These results suggest that the pairs simply preferred the lowest and highest in the F-major chord.

### Appendix A.2. Other Recurrence Metrics on Music

In addition to symbolic-recurrence rate and entropy, we examined other metrics on recurrence that could provide additional information about the structural features of music. These metrics are visualized over the aggregated symbolic-recurrence plot, where we only tracked the presence of recurrent behavior without book-keeping the specific partition of the phase space that was recurring.

In symbolic-recurrence plots, the presence of lines is indicative of the dynamic behavior of the system [24,25]. Diagonal lines of length *d* between (t,s) and (t+d,s+d) represent a sequence of symbols of which phase-space vectors $\left({\bar{x}}_{t},{\bar{x}}_{s}\right),\left({\bar{x}}_{t+1},{\bar{x}}_{s+1}\right),\ldots ,\left({\bar{x}}_{t+d},{\bar{x}}_{s+d}\right)$ belonged to the same set in the partition of the phase space. For example, should we have a diagonal line of length 2 between (t,s) and (t+2,s+2), we would infer that the ordinal patterns in the time series at t,t+1, and t+2 recurred at s,s+1, and s+2. Therefore, these lines identify deterministic processes of the system, where long lines indicate cyclical sequences [24].

Vertical lines (and horizontal ones for symmetry) of length *v* correspond to a sequence of phase-space vectors ${\bar{x}}_{t},{\bar{x}}_{t+1},\ldots ,{\bar{x}}_{t+v}$. For example, should we have a vertical line of length 2 at time *t*, we would infer that the ordinal pattern at time *t* would again be observed at times t+1 and t+2. Therefore, these lines identify laminar phases of the system, where long lines indicate persistent periods of the same states [24].

For each music piece created by the pairs, we counted the numbers of the diagonal lines of length *d* (d≥2) and the vertical lines of length *v* (v≥2), respectively. From the distributions of these lengths, we obtained a mean and maximal length of the lines. We also quantified the proportions of recurrences that formed diagonal and vertical lines, so-called determinism and laminarity, respectively [60]. The main diagonal line was excluded from quantification.

To test whether these metrics were different from chance, we compared the observed mean of the 30 pairs against the null distributions of the corresponding values for each metric (two-tailed permutation test). The null distributions were obtained by randomly shuffling partners and merging their individual sound data of a new pair. We obtained 20,000 means of each metric.

The metrics on the diagonal lines indicated the presence of many short cyclic patterns (Figure A2). In the first session, the diagonal lines were shorter than chance in the maximal length (p<0.001), with marginal significance with respect to mean length (p=0.064). Determinism was marginally greater than chance (p=0.057). By contrast, these metrics were not significantly different from chance in the second session (p=0.233 for mean, p=0.454 for maximum, and p=0.108 for determinism).

Similarly, vertical lines showed the presence of many short events of persistent periods (Figure A3). In the first session, the mean and maximal lengths were significantly shorter than chance (p=0.044 and p<0.001, respectively), but they were not different from chance in the second session (p=0.452 and p=0.481, respectively). Laminarity was not significantly different from chance in the first session (p=0.224), but it was greater than chance in the second session (p=0.040).

### Appendix A.3. Effect of Downsampling Rate on Recurrence Metrics

To assess the robustness of recurrence metrics, in the main manuscript we investigated changes in downsampling rate; we performed the same computation at a faster downsampling rate of 100 ms.This value should be considered a lower bound for human ability to respond to acoustic cues [61]. We symbolized the downsampled time-series with m=3, and reanalyzed the significance of each metric (SRR, entropy, mutual information, and transfer entropy) by comparing mean values against the null distribution generated from 20,000 permutation (one-tailed). Agreement with respect to inferences at a downsampling rate of 150 ms was evaluated using a Pearson correlation test in base R ver. 3.6.0 [32]. Prior to the test, mutual information and transfer entropy were log-transformed to normalize distribution.

All metrics were significantly different from chance in the same direction as the corresponding metrics obtained by downsampling the time series at a 150 ms interval (p<0.001 for all; Figure A4). Further, all metrics were positively correlated with the corresponding metrics computed from a downsampling rate of 150 ms (Figure A5). For SRR, the correlation coefficient was 0.841 in the first session (t=8.227,df=28,p<0.001) and 0.856 in the second session (t=8.745,df=28,p<0.001). For entropy, the correlation coefficient was 0.859 in the first session (t=8.864,df=28,p<0.001) and 0.854 in the second session (t=8.671,df=28,p<0.001). For mutual information, the correlation coefficient was 0.953 in the first session (t=16.556,df=28,p<0.001) and 0.865 in the second session (t=9.126,df=28,p<0.001). Finally, for transfer entropy, the correlation coefficient was 0.747 in the first session (t=8.554,df=58,p<0.001) and 0.745 in the second session (t=8.509,df=58,p<0.001).

### Appendix A.4. Effect of m on Recurrence Metrics

To test the robustness of our predictions with respect to the symbolization scheme, we evaluated the recurrence metrics by symbolizing the time series with a lower value of *m*, that is, m=2. This symbolization scheme generated two symbols on the basis of the dynamics of two consecutive samples (increasing and decreasing), thereby capturing less complexity in musical structures. Larger values of *m* are practically unfeasible due to the length of the available time series. We obtained each recurrence metric (SRR, entropy, mutual information, and transfer entropy) by converting the time series of sound amplitudes into two symbols. The significance of each recurrence metric was tested by comparing the mean value against the null distribution generated from 20,000 permutation (one-tailed). Agreement with respect to inferences with m=3 was evaluated using a Pearson correlation test in base R ver. 3.6.0 [32]. Prior to the test, mutual information and transfer entropy were log-transformed to normalize distribution.

All metrics were significantly different from chance in the same direction as the corresponding metrics obtained by symbolizing the time series with m=3 (p<0.001 for all; Figure A6). Further, all metrics were positively correlated with the corresponding metrics computed with m=3 (Figure A7). For SRR, the correlation coefficient was 0.920 in the first session (t=12.395,df=28,p<0.001) and 0.834 in the second session (t=7.999,df=28,p<0.001). For entropy, the correlation coefficient was 0.928 in the first session (t=13.187,df=28,p<0.001) and 0.870 in the second session (t=9.355,df=28,p<0.001). For mutual information, the correlation coefficient was 0.684 in the first session (t=4.961,df=28,p<0.001) and 0.698 in the second session (t=5.164,df=28,p<0.001). Finally, for transfer entropy, the correlation coefficient was 0.276 in the first session (t=2.184,df=58,p=0.033) and 0.262 in the second session (t=2.070,df=58,p=0.043).
